# Tumor-Associated Tertiary Lymphoid Structures: From Basic and Clinical Knowledge to Therapeutic Manipulation

**DOI:** 10.3389/fimmu.2021.698604

**Published:** 2021-06-30

**Authors:** Charlotte Domblides, Juliette Rochefort, Clémence Riffard, Marylou Panouillot, Géraldine Lescaille, Jean-Luc Teillaud, Véronique Mateo, Marie-Caroline Dieu-Nosjean

**Affiliations:** ^1^ Faculté de Médecine Sorbonne Université, Sorbonne Université, UMRS 1135, Paris, France; ^2^ Faculté de Médecine Sorbonne Université, INSERM U1135, Paris, France; ^3^ Laboratory “Immune microenvironment and immunotherapy”, Centre d’Immunologie et des Maladies Infectieuses Paris (CIMI-Paris), Paris, France; ^4^ Université de Paris, Faculté de Santé, UFR Odontologie, Paris, France; ^5^ Service Odontologie, Assistance Publique Hôpitaux de Paris (AP-HP), La Pitié-Salpêtrière, Paris, France

**Keywords:** artificial intelligence, biomarker, cancer, lymphoid neogenesis, organoid, tertiary lymphoid structure, therapeutic intervention, tumor model

## Abstract

The tumor microenvironment is a complex ecosystem almost unique to each patient. Most of available therapies target tumor cells according to their molecular characteristics, angiogenesis or immune cells involved in tumor immune-surveillance. Unfortunately, only a limited number of patients benefit in the long-term of these treatments that are often associated with relapses, in spite of the remarkable progress obtained with the advent of immune checkpoint inhibitors (ICP). The presence of “hot” tumors is a determining parameter for selecting therapies targeting the patient immunity, even though some of them still do not respond to treatment. In human studies, an in-depth analysis of the organization and interactions of tumor-infiltrating immune cells has revealed the presence of an ectopic lymphoid organization termed tertiary lymphoid structures (TLS) in a large number of tumors. Their marked similarity to secondary lymphoid organs has suggested that TLS are an “anti-tumor school” and an “antibody factory” to fight malignant cells. They are effectively associated with long-term survival in most solid tumors, and their presence has been recently shown to predict response to ICP inhibitors. This review discusses the relationship between TLS and the molecular characteristics of tumors and the presence of oncogenic viruses, as well as their role when targeted therapies are used. Also, we present some aspects of TLS biology in non-tumor inflammatory diseases and discuss the putative common characteristics that they share with tumor-associated TLS. A detailed overview of the different pre-clinical models available to investigate TLS function and neogenesis is also presented. Finally, new approaches aimed at a better understanding of the role and function of TLS such as the use of spheroids and organoids and of artificial intelligence algorithms, are also discussed. In conclusion, increasing our knowledge on TLS will undoubtedly improve prognostic prediction and treatment selection in cancer patients with key consequences for the next generation immunotherapy.

## Introduction

The study of tertiary lymphoid structure (TLS) formation, cellular content and function in tumors has progressed tremendously since the initial discovery of their presence in non-small cell lung carcinoma (NSCLC) and their association with more favorable clinical patient outcome (1). Numerous investigations on TLS have been performed with tumor biopsies, using immunohistochemical (IHC), and immunofluorescence (IF) labeling techniques and molecular analyses (transcriptomic, RNAseq, proteomic) leading to an increased knowledge of their neogenesis, composition, and immune functions (2). In particular, study of tumor biopsies has made possible to highlight the importance of tumor-infiltrating leucocytes (TIL) i.e., effector CD8^+^ T cells and TLS-B cells with regard to the clinical outcome (3, 4) and response to anti-immune checkpoint therapies (5–7). In the present review, we will first detail how TLS are potent anti-tumor structures associated in most cases with better prognosis of cancer patients and how they can be boosters of anti-tumor responses elicited by anti-ICP (immune checkpoint) immunotherapy. We will then examine their relationship with tumor genetic instability, oncogenic drivers in cancer patients, and oncogenic viruses, with a particular focus on NSCLC and Head and Neck Squamous Cell Carcinoma (HNSCC). Second, we will discuss some aspects of TLS neogenesis and function learned from inflammatory diseases and we will present preclinical studies performed in tumor models that suggest that TLS manipulation could represent a potent new anti-cancer immunotherapy.

## Importance of the Organization of Tumor-Infiltrating Immune Cells in Tumor Immunosurveillance

The efficacy of immunotherapy in cancer relies on the generation of an efficient and long-lasting adaptive immune anti-tumor response. However, objective response rates remain low, between 20% to 40%, due to tumor immune escape and to a lack of accurate predictive biomarkers (8). Furthermore, immune-related adverse events occur because of enhanced T cell activation, leading sometimes to treatment discontinuation (9). Thus, there is a crucial need to discover new immune pathways that can be manipulated to improve responses to immunotherapies and cancer patient prognosis.

**Figure 1:**
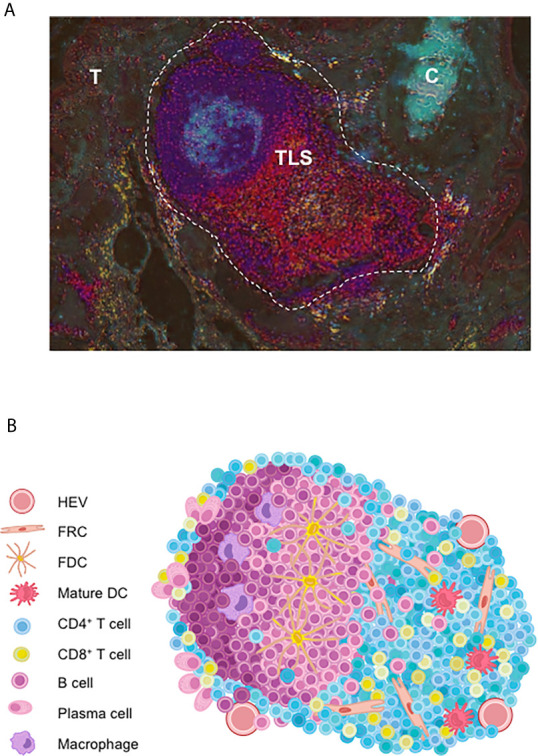
Cellular organization of tumor-associated TLS. **(A)** Full TLS image of lung tumor sections in a NSCLC patient. Briefly, two serial tumor sections were respectively double-immunostained for CD3/DC-Lamp and CD20/CD21. Then color deconvolution and contrast inversion (using ImageJ) was applied as described (10). Shown are CD3^+^ T cell-rich areas (red); DC-Lamp^+^ mature DC (yellow) adjacent to the CD20^+^ B-cell rich areas (dark blue) and CD21^+^ FDC (light blue) of a TLS close to tumor nests (T) and bronchial cartilage (C). Magnification: x100. **(B)** TLS are well-organized functional immune ectopic aggregates in the close vicinity to HEV. TLS comprise a T-cell zone containing mature DC and FRC; and a B-cell zone with a germinal center with PC, macrophages and FDC. C, bronchial cartilage; DC, dendritic cell; FDC, follicular dendritic cell; FRC, fibroblastic reticular cell; HEV, high endothelial venule; PC, plasma cell; T, tumor nest.

A number of reports highlights growing evidence on that the development of anti-tumor immune responses at the tumor sites within organized lymphoid structures called TLS that act as local hubs where the immune response can be generated. Initially evidence in the context of autoimmune pathologies, TLS comprise the various cellular components needed to develop an adaptive anti-tumor response. They exhibit a T-cell zone containing activated T cells with Th1, cytotoxic or memory phenotypes, mature dendritic cells (DC) involved in antigen presentation, and fibroblastic reticular cells (FRC) (**Figures 1A, B**). A B-cell zone is also present, exhibiting a germinal center (GC) and where memory B cells and plasma cells can be detected (3, 4). On one hand, detailed cellular content and organization of ectopic lymphoid aggregates have been described, leading to the view that TLS neogenesis is a complex process that gives rise to different types of lymphoid aggregates until a fully differentiated TLS is generated. On the other hand, important features of TLS in cancer patients that have also brought new insights in their relationship with clinical status and tumor characteristics, as described below.

### Prognostic Impact of Tumor-Associated TLS

TLS have been observed in numerous tumor types such as NSCLC, HNSCC, ovarian cancer, breast cancer, hepatocellular carcinoma (HCC) or gastrointestinal stromal tumor (GIST). High densities of TLS are associated with better relapse-free survival (RFS) and overall survival (OS) in several types of solid cancer, independently of tumor TNM staging which is considered as the most important prognostic factor in cancers. The prognostic impact of TLS has been largely reviewed, and it has been shown that, alike in NSCLC, a more favorable outcome for patients is observed in a large number of other cancer types (11). Notably, the high density of TLS in OSCC patients has been associated with a better OS and RFS (12) and identified as an independent positive prognostic factor (13). However in HCC, their prognostic value remains a matter of debate with the description of a poor *versus* a favorable clinical outcome (14, 15), and HCC risk factors such as alcohol consumption, HCV and HBV infection do not account for this discrepancy as these parameters are correlated with TLS densities. Of note, regulatory T lymphocytes (Treg) have been observed in lymphoid aggregates [breast tumors (16)], and TLS [breast cancer, lung SCC, prostate cancer and lung metastasis (17–20)], and their high densities have been associated with a poor clinical outcome suggesting an immunosuppressive role of Treg in these ectopic lymphoid organizations.

Other studies also took into consideration the status of TLS maturation within the tumors, from an immature stage i.e., dense lymphoid aggregates without a network of follicular dendritic cells (FDC), to fully a mature TLS with the segregation of T and B cells segregated into two distinct areas. Thus, immature TLS are present in dysplastic nodules at a pre-neoplastic stage of HCC (21) and in colorectal carcinoma (CRC) (22), and correlate with an increased risk of cancer relapse. Thus, if it is agreed that lymphoid aggregates are immature TLS, this very early stage of TLS development appears to be unable to promote an efficient anti-tumor immune response. A higher level of TLS organization is mandatory to reach a more sophisticated structure allowing an optimal dialogue between the different actors of immune responses, namely T and B cells, macrophages, DC, and FDC (**Figure 1B**).

In addition until now, the relationship between the prognostic value of TLS and their *in situ* localization in adjacent non-tumor tissue it is still a matter of debate. TLS located in distant non-tumor tissue have been associated either with an increased rate of relapse (14), or no value in HCC (15). In contrast in breast cancer, a negative prognostic value has been reported when TLS are present in peri-tumor tissue while intra-tumor TLS are mainly associated with a favorable outcome (23). However, TLS were defined by a chemokine gene signature or by hematoxylin/eosine counterstaining in these studies, and further investigation are required to define the maturation stage of these lymphoid organizations. Thus, the localization of TLS with regard to tumor masses seems to be critical. It underlines the importance of defining the invasive margin for investigating the role of TLS in solid tumors.

Finally, TLS anti-tumor efficacy may also be dependent on tumor stage and on tumor sites where they are located. In melanoma, TLS are found in metastatic sites but not in primary sites (24), although one has to stress that it is difficult to identify primary tumors in most melanoma patients. Lung metastases from renal cell carcinoma (RCC) exhibit mostly immature TLS and correlate with short-term survival whereas in CRC lung metastases, TLS are more mature and are associated with a favorable outcome even at very advanced stage of the disease. Notably, their density was similar between the primary and their matched metastases (25). Thus, these data suggest that the tumor origin seems to be very critical in the shaping of a peculiar immune environment where TLS neogenesis can occur - or not, as compared with the metastatic sites.

### Interplay Between TLS and Anti-Cancer Therapies

**Figure 2:**
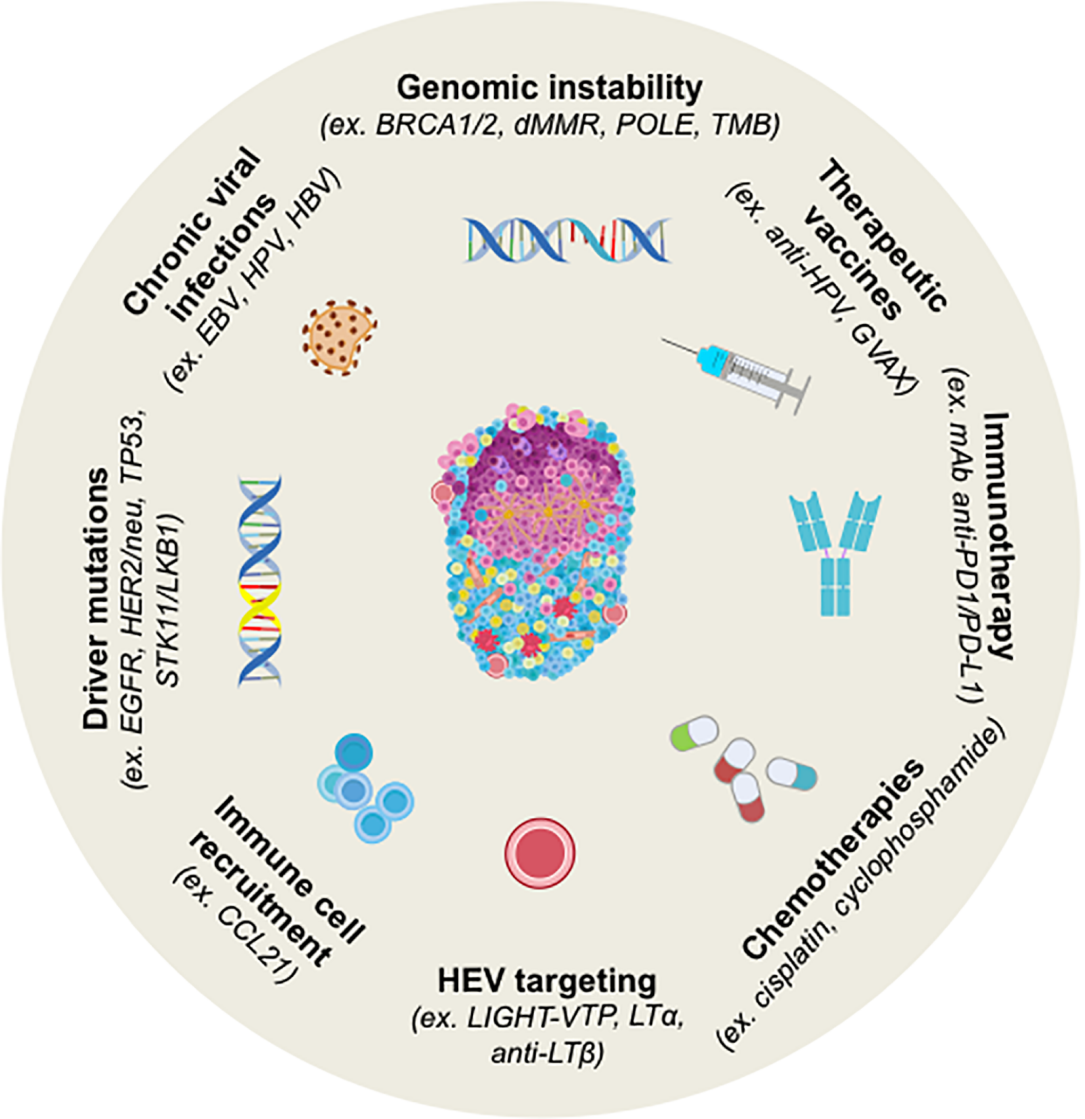
Strategy for Induction of TLS neogenesis in human cancers and by cancer treatments. TLS are induced by chronic viral infections to which tumorigenesis has been associated with genomic instability and/or peculiar driver mutations in several tumors. Chemotherapy, immunotherapy or therapeutic vaccination can also induce TLS. In most cases, their presence is concomitant to better prognosis and higher clinical responses to treatments. Murine models have been explored for the induction of TLS by targeting HEV or the cells involved in organogenesis (i.e. CCL21). BRCA1/2, Breast cancer 1 and 2; dMMR, Deficient Mismatch Repair; EBV, Epstein-Barr Virus; EGFR, Epidermal Growth Factor Receptor; GVAX, GM-CSF-secreting allogeneic PDAC vaccine; HPV, Human Papillomavirus; LIGHT-VTP, LIGHT (stands for *homologous to lymphotoxin*)-Vascular Targeting Peptide; LKB1, Liver kinase B1; LTα, lymphotoxin alpha; LTβ, lymphotoxin beta; OSCC, Oral Squamous Cell Carcinoma; PDAC, pancreatic ductal adenocarcinoma; POLE, DNA Polymerase Epsilon, Catalytic Subunit; STK11, Serine Threonine Kinase 11; TMB, Tumor Mutational Burden.

TLS are increasingly considered as a predictive biomarker of responses to anti-cancer therapies such as chemotherapy, immunotherapy, or targeted therapy (**Figure 2**). It is likely related to the induction of an immunogenic cell death (ICD) that leads to the release of neo-antigens that are then captured by DC, triggering an anti-tumor immune response. In triple-negative breast cancer (TNBC), the high density of high endothelial venules (HEV, as a surrogate marker of TLS) correlates with the pathologic complete response (pCR) after neo-adjuvant chemotherapy (26). In addition, the presence of TLS could also predict a better outcome in patients treated with targeted therapies. Treatment of *HER2/neu* ^+^ tumors with trastuzumab, a monoclonal antibody (mAb) targeting HER2/neu, has been associated with a better disease-free survival in TLS-enriched tumors (27). In gastrointestinal stromal tumors (GIST), a high density of TLS has been associated with lower imatinib resistance, recurrence, and a more favorable survival (28). Finally, the cellular composition of TLS was found to be different in imatinib-resistant *versus* non-resistant patients, with more regulatory T cells in resistant GIST.

Anti-cancer immunotherapy has been proven one of the most important therapeutic advances in cancer treatment over the last few decade. However, all patients do not respond to these treatments, and there is a crucial need to determine accurate predictive biomarkers to better stratify patients. TLS could be one efficient biomarker. The presence and density of TLS have been correlated with responses to immune checkpoint (ICP) therapies in clear cell renal cell carcinoma (ccRCC) treated with anti-PD1 antibody, or in melanoma treated with a combination of anti-PD1 and anti-CTLA-4 antibodies (5, 6). Similarly, in soft-tissue sarcoma, the presence of TLS and B cells has been also correlated with ICP responses (7). Once again, the maturation stage of TLS and their cellular content appear to correlate their ability to represent a predictive biomarker. In the NABUCCO trial in bladder cancer, the induction of TLS with anti-PD1 and anti-CTLA-4 combination correlated with ICP responses (29). Immature TLS were observed in most non-responding patients, pinpointing again the important role of the maturation status of TLS. Also, one of the main issues of ICP treatment is the occurrence of immune-related adverse events (irAE) that sometimes leads to therapy discontinuation. While corticosteroid therapy received in response to irAE does not impair response to ICP treatment, it was reported that it dampens TLS formation and maturation in lung squamous cell carcinoma, most probably because TLS maintenance is dependent on inflammation (30).

In hepatoblastoma with *APC* mutations, an increase in TLS formation probably favored by ICD has been observed in paired biopsies from pre- and post-cisplatin-based chemotherapy (31). Similarly, NSCLC patients treated with anti-PD1 antibody in a neo-adjuvant setting showed an enrichment in TLS (32), with a positive correlation with higher response rates (33). Vaccine-based immunotherapy can also induce TLS formation (**Figure 2**). The induction of TLS upon HPV vaccination has been observed in responding patients with cervical neoplasia and correlated with CD8^+^ T cell and Th1 infiltration (34). Moreover, it has been shown that irradiated GM-CSF-secreting allogeneic PDAC vaccine (GVAX) could switch a non-immunogenic to an immunogenic tumor in pancreatic ductal adenocarcinoma (PDAC) patients, marked by a strong T cell infiltration and TLS neogenesis (35). In line with these results, several murine models of cancer have demonstrated that induction of TLS through different strategies can overcome resistance to ICP blockade and synergize with ICP therapy (see *Learning From TLS Study in Non-Tumor Inflammatory Diseases to Better Understand TLS Role in Cancer*).

### Genomic Instability, Impaired DNA Repair and TLS: A Relationship to Investigate

Due to genomic instability and impaired reparation processes, tumor cells accumulate genetic abnormalities. Few reports have focused on the molecular characteristics of tumors with regard to TLS presence and function. So far, the relationship between tumor mutational burden (TMB) and TLS in cancers has been rarely investigated (**Table 1** and **Figure 2**). Based on a 12-chemokine signature using the TCGA database, Lin et al. reported that tumors having high TMB exhibit high TLS densities in NSCLC and melanoma, two cancers types known to respond to ICP (38). This highlights the importance of assessing other molecular and cellular signatures in addition to TLS density to predict responses to cancer therapies.

**Table 1:** Correlation of TLS signature with driver gene mutations in human cancers.

Mutational status	Read-out	Tumor type	Number of patients	Method of TLS detection	Correlation with mutational status	Ref.
BRCA1/2	protein	TNBC	85	IHC CD3/CD20	No correlation between TLS and BRCA-mutational status	(36)
					High PD-1 expression on immune cells within TLS compared with stromal immune cells	
					Higher prevalence of PD-L1 positive tumors within the TLS positive tumors	
		High-grade serous ovarian cancer	30	IHC CD3-CD8-CD20-CD21-CD208-PNAd	Correlation between TLS and TIL infiltration	(37)
	transcriptomic	Breast cancer, prostate ADC, and CESC	1119, 502, and 306	TCGA database	TLS signature correlates with BRCA1/2 for breast cancer, prostate adenocarcinoma and endometrial carcinoma	(38)
		High-grade serous ovarian cancer	30	TCGA database	Plasma cell or B cell signature correlate with TIL but not with BRCA1/2 status	(37)
MSI	protein	Colorectal cancer (stages II/III)	109	IF CD20	Higher TLS formation with MSI status	(22)
					Higher maturation rate of TLS with MSI status	
	transcriptomic	CESC	306	TCGA database	TLS signature correlates with MSI status	(38)
		Endometrial carcinoma	119	TCGA database	TLS signature correlates with MSI status	(39)
		Colorectal cancer	975	12-chemokine transcriptomic signature	MSI status correlates with higher TLS signature	(40)
CIMP	transcriptomic	Colorectal cancer	975	12-chemokine transcriptomic signature	CIMP status associated with higher TLS signature	(40)
POLE	protein	Endometrial carcinoma	119	IF CD20, CD3, CD8, CD11c, PNAd	TLS signature correlates with POLE mutation	(39)
	transcriptomic	CESC	306	TCGA database	Trend for higher TLS signature for POLE	(38)
TMB	transcriptomic	All tumors	8672	TCGA database	TLS scoring correlates with neo-antigen burden for bladder, breast, cervical, lung adenocarcinoma, endometrial and stomach	(38)
EGFR	protein	NSCLC (ADC)	221 + 24 + 32	IHC CD3/DC-Lamp	EGFR mutations highly represented in TLS^high^ patients	(41)
EGFR	protein	NSCLC (ADC)	316	IHC CD8/DC-Lamp	Trend for higher EGFR mutations in TLS^high^ patients	(42)
Her2	protein	Breast cancer	248	IHC CD3/CD20/CD23	more TLS in HER2^+^ compared with Her2^-^	(43)
					TLS associated with higher TIL infiltrate	
		Breast cancer	95 (32/31/19/13)	IHC CD3/CD20	PD-1^high^ TIL most often found in TLS^high^ tumors (frequently TNBC and HER2^+^ tumors)	(44)
		Breast cancer	447 HER2^+^ (HR^+^ or HR^-^)	HES	TLS density correlates with HER2 expression modification	(27)
KRAS	protein	NSCLC (ADC)	221 + 24 + 32	IHC CD3/DC-Lamp	no association with KRAS mutations	(41)
BRAF	protein	NSCLC (ADC)	221 + 24 + 32	IHC CD3/DC-Lamp	no association with BRAF mutations	(41)
		Colorectal cancer (stages II/III)	109	IF CD20	Positive correlation between BRAF mutations high TLS scoring	(22)
					BRAF mutations correlate with presence of higher mature TLS	
		Colorectal cancer (stages II/III)	351	IHC CD3/CD20	No correlation between TLS density and MSI status	(45)
		NSCLC (ADC)	316	IHC CD8/DC-Lamp	BRAF mutations correlate with low CD8^+^ T cell and mature DC infiltrate	(42)
	transcriptomic	Colorectal cancer	975	12-chemokine transcriptomic signature	High TLS status correlates with right-sided tumor, BRAF mutations, and MSI-high status	(40)
					BRAF mutations correlate with high TLS signature	
STK11	protein	NSCLC (ADC)	221 + 24 + 32	IHC CD3/DC-Lamp	STK11 mutations correlate with low infiltration of T cells and mature DC	(42)
		NSCLC (ADC)	316	IHC CD8/DC-Lamp	STK11 mutations correlated with low infiltration of CD8^+^ T cells and mature DC	(42)
TP53	protein	NSCLC (ADC)	221 + 24 + 32	IHC CD3/DC-Lamp	TP53 mutations correlate with high T cell and mature DC infiltrate	(41)
		HNSCC	65	IHC CD3/CD20/CD21	TLS density associated with decreased P53 mutations	(46)
	transcriptomic	Colorectal cancer	975	12-chemokine transcriptomic signature	TP53 wt correlates with lower TLS signature in one cohort of patients	(40)
		All tumors	8672	TCGA database	Positive correlation between TLS scoring and TP53 mutations in breast cancer and low grade glioma	(38)
					Negative correlation between TLS scoring and TP53 mutations in HNSCC and stomach cancer	
					No correlation between TLS scoring and lung cancers	
EBV	transcriptomic	Gastric cancer	420	TCGA database	Positive correlation between TLS signature and EBV infection	(38)
HPV	transcriptomic	HNSCC	504	TCGA database	Positive correlation between TLS signature and HPV infection	(38)
		CESC	306	TCGA database	No correlation between TLS signature and HPV infection	
HBV	transcriptomic	HCC	374	TCGA database	No correlation between TLS signature and HBV infection	(38)

Several methods have been used for the quantification of TLS, as discussed in (11).

ADC, Adenocarcinoma; CESC, Cervical Squamous cell Carcinoma and Endocervical adenocarcinoma; EBV, Epstein-Barr virus; HBV, Hepatitis B virus; HCC, HepatoCellular Carcinoma; HNSCC, Head and Neck Squamous Cell Carcinoma; HPV, Human Papilloma virus; IF, ImmunoFluorescence (staining); IHC, ImmunoHistoChemistry; MSI, Microsatellite Instability; NSCLC, Non-Small Cell Lung Carcinoma; TCGA, The Cancer Genome Atlas; TIL, Tumor-infiltrating Leukocyte; TNBC, Triple Negative Breast cancer; TMB, Tumor Mutational Burden; wt, wild type.

Tumors are highly instable and harbor high rates of mutations leading to the expression of neo-antigens that in turn could trigger anti-tumor immune responses. This is due, at least in part, to an impairment of the DNA repair machinery through defects in homologous recombination (HR) DNA repair genes. The gene encoding *BRCA* that is involved in the detection and reparation of DNA alterations, is mutated in tumors such as breast or ovarian cancers. *BRCA*-mutated tumors exhibit a strong infiltration by CD8^+^ T cells that harbor a high PD-1 expression in ovarian cancer and some breast cancers such as HER2/neu^+^ tumors and TNBC (44, 47). Whereas patients with breast cancer poorly respond as well to ICP, patients with TNBC harbors response rates under ICP between 15% and 20% following ICP monotherapy and up to 58% when associated with chemotherapy (48). However, data regarding the putative link between *BRCA* mutations and TLS formation are scarce. Willard-Gallo and colleagues found no difference in the density, location or composition of TLS between *BRCA*-mutated and non-mutated TNBC in spite of a higher rate of TIL and PD-1 expression in TLS in *BRCA*-mutated tumors (36). In high-grade serous ovarian carcinoma, Nelson and colleagues did not find any correlation between a B-cell gene signature (as a likely hallmark of TLS presence (4–7), and *BRCA* mutations (37). By *in silico* analysis based on the TCGA database, Lin *et al.* elegantly showed a correlation between TLS scoring and mutation/neo-antigen loads in many solid tumors such as NSCLC, stomach adenocarcinoma, and uterine corpus endometrial carcinoma but not in others such as ovarian cancer (38). Many mutations as *BRCA* mutations positively correlate with high densities of TLS in most tumors including breast, prostate or endometrial cancers. Other mutations in genes such as *CTNNB1* and *IDH1* negatively correlate with high TLS scoring. Importantly, the correlation can be either positive or negative depending on the tumor type, as for *PIK3R1*. No common molecular feature was observed between HNSCC, lung adenocarcinoma and lung SCC that share some common risk factors. In HNSCC, TLS scoring correlates positively with *CASP8, EP300*, and *KMT2C* mutations, and negatively with *TP53* and *KMT2D* mutations. Similarly, a positive correlation was reported for *KEAP1* and *SMAD4* in lung ADC whereas a negative correlation was observed for *PIK3R1* in lung ADC and for *FBXW7* and *KMT2C* in lung SCC. Also, polymerase ϵ (POLE) mutations in endometrial carcinoma correlate with TLS presence (38). POLE is an enzyme involved in repair mechanisms such as base- or nucleotide-excision repair or homologous recombination, and mutations lead to hypermutations within tumors.

Moreover, the alterations of the DNA mismatch repair (MMR) system induce microsatellite instability (MSI), a condition of genome hypermutability. MSI are found in several types of cancers, such as CRC and endometrial cancers. In clinical practice, MSI tumors are associated with high response rates to PD-1 blockade (approximately 50%) whereas microsatellite stable (MSS) CRC are unresponsiveness (49). In stages II/III CRC, TLS and especially mature TLS are increased in MSI positive tumors and correlate with a better outcome compared with immature TLS (22, 50). Similar results were obtained using a 12-chemokine signature on fixed tissues or through TCGA database rather than immunohistochemistry as hallmark of TLS (38, 40), indicating that different approaches can be used for TLS quantification.

### Oncogenic Drivers, TLS, and Targeted Therapies

Specific oncogenic drivers i.e., mutated molecules involved in cell proliferation, activation and survival, have been defined in many cancers. This has led to the development of targeted therapies. For instance, different response rates to ICP treatments have been observed in cancerous patients depending on the genetic profile of the tumor. In NSCLC, the presence of *EGFR* mutations is associated with a lower response rate and a worse outcome under ICP whereas a high TMB is correlated with a better response, making TMB a predictive biomarker of ICP response (51, 52). In addition, some mutations are closely associated with a particular immune profile (exclusive, “cold” or “hot” tumors) and TLS scoring (**Table 1**). In NSCLC, *EGFR*-mutated tumors respond to tyrosine kinase inhibitors such as osimertinib, but they do not respond to immunotherapy most likely due to a low immune infiltrate. Conversely, *KRAS* mutations are associated with a higher immune infiltration and *KRAS*-mutated tumors classically respond to ICP, due to high TMB induced by tobacco exposure. In lung adenocarcinoma, Biton et al. have reported that the frequency of *EGFR* mutations is higher in tumors enriched with mature DC (as a hallmark of TLS) whereas no correlation has been found with *KRAS*, *TP53 and BRAF* mutations (41).

Mutations of *BRAF* are detected in several solid cancers, such as NSCLC, melanoma, and colorectal cancers. In CRC, *BRAF* mutations are associated with a higher TLS density, and TLS are more mature compared with *BRAF* wild type (wt) tumors (22, 40). Surprisingly, little is known regarding the relationship between *BRAF* mutations and TLS presence in melanoma, although patients have benefited from anti-BRAF targeted therapies for nearly ten years. Only one study has mentioned an absence of any correlation between B-cell transcriptomic profile (as TLS signature) and *BRAF* mutations in melanoma patients treated by neo-adjuvant targeted therapy (6). Thus, the apparent opposite observations seen in CRC and melanoma means that, in addition to *BRAF* mutations, other intrinsic parameters might be involved in the shaping of the tumor microenvironment (TME) including the development of TLS.

As discussed above, ICP response depends on various parameters among which *TP53* and *STK11* mutations. In NSCLC, *TP53* mutations are associated with better ICP responses whereas *STK11* mutations are correlated with resistance to the treatment (53). No correlation was observed between *TP53* mutations and TLS scoring in NSCLC although *TP53*-mutated tumors have a higher CD8^+^ T cell infiltrate and PD-L1 expression (38, 41). Conversely, *STK11*-mutated tumors are characterized by a lower infiltration of CD8^+^ T cells and TLS-mature DC infiltrate and a lower PD-L1 expression compared with *STK11*-wt tumors. Overall, the different response rates to ICP observed in NSCLC patients with either *TP53* or *STK11*-mutated tumors might be related to the composition and organization of the TME. In particular, patients with *STK11*-mutated lung tumors are not prone to respond to any ICP (at least targeting effector cells and PD-1/PD-L1 axis) as they have a marked deficit in TLS densities and PD-L1 expression.

### Cancer-Induced Viruses and TLS

Some murine models have elegantly demonstrated that chronic viral infection (e.g. Influenza virus) can elicit the neogenesis of TLS. Among them, some viruses can cause cancers. Thus, an open question is to determine whether the presence of a virus might be beneficial for the host as a source of foreign antigens, or harmful because of its oncogenic properties (**Figure 2** and **Table 1**). The most studied oncovirus is the human Papilloma virus (HPV) which is involved in several types of cancers such as HNSCC, cervical carcinoma, and anal carcinoma. Transcriptomic analysis using the TCGA database has shown a positive correlation between high TLS scoring and HPV^+^ HNSCC whereas no association was observed in cervical cancer (38). In gastric cancer, a high TLS signature was observed in the vast majority of EBV^+^ tumors whereas it was highly heterogeneous among EBV^-^ tumors. Finally, hepatitis B virus (HBV) infection was not associated with TLS scoring in liver cancer. All together, these results indicate that the ability of an oncovirus to induce TLS formation is dependent on the type of cancer and its TME. Further investigation is required to decipher the mechanism by which a virus can favor – or not - the initiation of TLS neogenesis, and to evaluate whether anti-viral immune responses can take place in TLS. Such approaches could give rise to the identification of a new category of TLS-inducer candidates for therapeutic purposes.

## Learning from TLS Study in Non-Tumor Inflammatory Diseases to Better Understand TLS Role in Cancer

Long before they draw any interest in oncology, TLS were initially described in diseases where chronic inflammation is a shared feature. They were observed in organs that are the targets of autoimmune effector mechanisms and during solid organ transplant rejection, in some chronic infections by pathogens (bacteria and viruses), and in allergy (54–57). In autoimmune diseases and allograft rejection, their presence has been associated with an unfavorable clinical evolution (54–58), as opposed to the favorable outcome observed in cancers associated with their presence. In local infections, their role and prognostic value are more variable and depend on their location, composition and on the considered pathogen (58, 59). Remarkably, the study of TLS in these pathological conditions has provided a better understanding of the events that lead to their neogenesis in specific organs and might give some helpful clues in the field of tumor immunology (57).

In fact, as for tumor-associated TLS, tissue-related TLS also share many features with secondary lymphoid organs. Scrutiny of TLS induction in non-tumor bearing animal models has provided with crucial information for the understanding of the complex, tightly regulated, non-redundant mechanisms that lead to initiation, formation and maintenance of these lymphoid ectopic structures (55). Notably, several recent reports have shed new light in the ontogeny of TLS in mouse models of lung disorders (33, 60–66). In a model of viral infection with respiratory syncytial virus (RSV), Gassen et al. reported a RSV-dependent down-modulation of both IL-21 and IL-21R expression by lung Tfh cells, accompanied by an up-regulation of PD‐L1 expression on resident B cells and DC, resulting in a defective GC formation and anti-RSV antibody response. Therapeutic blockade of PD‐L1 restored IL‐21R expression and increased IL‐21 secretion by Tfh cells. In parallel, treatment of RSV-infected mice with IL‐21 decreased the viral load and inflammation, while inducing the formation of TLS and improving the antibody response against RSV in the lung of infected animals (60). These results highlight the complex interplay between the IL‐21/IL‐21R and the PD‐1/PD‐L1 axes leading to the regulation of Tfh function and TLS formation in RSV-infected lungs. This is reminiscent of the observations made in NSCLC, where the presence of tumor-associated TLS is a favorable factor for an objective response to treatment with PD-1/PD-L1 blocking antibodies (32, 38). Thus, one can hypothesize that these anti-ICP therapies stimulate Tfh and the formation and/or maintenance of TLS in responder patients. Interestingly, an increase of IL-21 and IL21R transcripts and of IL-21 secretion is associated with the presence of TLS in Nasal Inverted Papilloma, a benign tumor, strengthening the idea that IL-21/IL21R axis plays a critical role in the response to anti-ICP therapy, possibly through the formation of TLS (67).

TLS are also involved in the control of lung bacterial infection (62, 66). In *Mycobacterium tuberculosis* (TB) infection, they have been associated with gender bias susceptibility to TB (68). More recently, the same authors demonstrated that premature death of males after TB infection is associated with smaller B-cell follicles in the lungs during the chronic phase of the infection. Moreover, the amount of IL-23, a cytokine required for IL-17 response to infection with TB was reduced in lungs of TB-susceptible males as compared with resistant females (69). This pinpoints an underestimated gender bias in TLS formation during TB infection (65). Of note, a higher proportion of males has been observed in TLS^low^ *versus* TLS^high^ NSCLC patients (3). It will be certainly important to evaluate in retrospective studies whether such a bias exists in other infectious and malignant diseases.

Chronic Obstructive Pulmonary Disease (COPD) is often associated with airway epithelium functional defects, including a lack of secreted IgA (sIgA) and a pathological adaptive immune activation in advanced COPD patients. Richmond et al. have reported that TLS-like structures (i.e., accumulation of dense B-cell aggregates surrounded by CD4^+^ and CD8^+^ T cells) and myeloid DC are present in the lungs of COPD patients where no sIgA are detected, as observed in sIgA-deficient mice (64). Thus, airway bacteria induce the migration of monocyte-derived DC in the lungs through a CCR2-dependent mechanism which in turn favor T cell recruitment and TLS formation.

By the meantime, Naessens et al. have studied the lung myeloid compartment in a COPD cohort using single-cell RNA sequencing and identified type 2 conventional DC (cDC2) as being the more abundant DC in COPD lungs. These authors could show that these cells exhibit a unique migratory signature, including transcripts encoding CXCR5, CXCR4, and the oxysterol receptor EBI2, known to control the spatial organization of cells within TLS (61). Moreover, COPD cDC2 strongly express OX40L, enabling the induction of Tfh cells through the OX40–OX40L axis. Interestingly, OX40L expression by abnormal accumulation of thymic B cells has also been reported to promote Tfh cells in TLS from lupus-prone BWF1 mice (70), pinpointing the importance of this co-stimulatory molecule on B cells for ectopic GC formation in autoimmune targeted organs (71). Importantly, the level of expression of OX40L in human glioblastoma has been associated with a better prognosis, and mice bearing OX40L-expressing glioblastoma present with an improved survival rate over OX40L negative tumors (72). As lymphoid tissue inducer (LTi) cells also express OX40L, the OX40/OX40L axis is therefore likely an interesting therapeutic target for cancer therapy with regard to TLS induction. Anyhow, whether OX40/OX40L axis is implicated in tumor TLS remains to be demonstrated, and current clinical trials testing OX40 agonists for several oncology indications may provide key answers (73). However, LTi cells may not be essential for TLS formation in some situations, as illustrated in mice devoid of LTi cells (74, 75). Several immune cells have been shown to activate local resident mesenchymal stromal cells through LTα and TNF-α receptors (see *The Use of Pre-Clinical Models for studying TLS: From Imaging to Therapeutic Manipulation*), a key cross-talk enabling TLS neogenesis (76).

In an elegant model of kidney injury, Luo et al. also reported a central role of IL-17 in TLS formation. The model has the advantage to allow for a long-lasting development and maintenance of TLS, as animal are sacrificed 45 days after having ischemia reperfusion kidney injury. In this setting, the generation of TLS required fibroblastic reticular cells (FRC) that produce large amount of CXCL13 and CCL19. Interestingly, the size of kidney TLS was dependent on the concentrations of these lymphoid chemokines. Remarkably, in a cohort of patients suffering from IgA nephropathy (IgAN) condition, of whom about 30% present with renal TLS, the authors have reported a significant higher plasma level of CXCL13, CCL21, and CCL19 in patients exhibiting kidney TLS, thus representing a non-invasive renal TLS biomarker (77).

The CCL21 and CXCR3 axis have also been found central when studying a model of local TLS formation during allogeneic aortic transplant chronic rejection. By single-cell RNA sequencing, the authors identified lymphatic endothelial cells as CCL21-producing cells, recruiting into the aortic transplant CXCR3^+^ and CCR7^+^ cells involved in TLS formation in arteriosclerotic vessels in the recipient animals (78), confirming previous observations made in mice and humans (79). The presence of TLS induced *via* CCL21 in these pathological conditions is associated with an unfavorable prognosis, and blockade of the CCL21 and CXCR3 axis improves disease conditions (78). Surprisingly, the presence of CCL21 may be also associated with decreased autoimmune symptoms. In a diabetic NOD mouse model, Badillo et al. have interrogated the impact of CCL21 on TLS formation in diabetic animals. When comparing TLS from wild-type (WT) NOD mice and animals expressing CCL21 (ins2-CCL21-NOD) under the control of insulin, the latter mice differ from inflammatory NOD TLS found in type 1 diabetic islets isolated from WT NOD mice. As opposed to the TLS observed in the WT NOD mice, these TLS resemble lymph nodes, contain FRC-like cells expressing β-cell auto-antigens, and are able to induce systemic and antigen-specific tolerance leading to diabetes prevention (80). Remarkably, the authors noticed a redistribution of Treg to ins2-CCL21-NOD islets, presumably through the expression of CCR7 on these regulatory cells. Thus, therapeutic manipulation of CCL21 in autoimmune conditions and cancer may represent a double-edged sword approach and certainly needs further investigation.

Overall, the exploration of mice models of non-malignant inflammatory diseases has provided valuable information on TLS neogenesis. As reviewed above, a number of cellular networks and pathways have been revealed as playing an essential role in the generation of functional TLS. It will undoubtedly help to better define molecular and cellular targets aimed at manipulating the formation and the function of TLS in various diseases, including cancers.

## The Use of Pre-Clinical Models for Studying TLS: From Imaging to Therapeutic Manipulation

Increasing attention has been given to TLS neogenesis in the local tumor microenvironment over the last two decades, due to their negative role in inflammatory/autoimmune diseases and graft rejection on the one hand, and to their positive association with clinical responses in some infectious diseases, cancers, and in response to anti-ICP therapy.

However, the study of biopsies has important limitation for exploring the role of the different cell subsets present in TLS, their circulation and their dynamic cognate interactions. In oncology, studies on human tumor biopsies derived from cancers such as NSCLC, have been limited in particular by a limited access to samples of sufficient sizes and, hence, the difficulty to perform *in vitro* functional studies with cells derived from these biopsies. To circumvent this bottleneck, one can take advantage of the technical advancements that have included i) the establishment of cell lines (a large majority being of human origin), ii) the use of primary cell cultures, and iii) the development of various mouse models (as illustrated in (81) for NSCLC).

This has led to the setting of preclinical tumor models to elucidate the cellular and molecular mechanisms underlying the formation and immune function of TLS. These studies go from easy-to-use syngeneic subcutaneous tumor transplants (which might fail at mimicking the relevant microenvironment conditions) to more sophisticated genetically engineered models, in which autochthonous tumors progress in the presence of a fully functional immune system, enabling the microenvironment changes and immune evolutionary adaptation that are requested for the spontaneous formation of TLS.

### Deciphering Mechanisms of TLS Neogenesis to Explore Their Therapeutic Manipulation in Murine Models

#### Syngeneic Tumor Models

**Figure 3:**
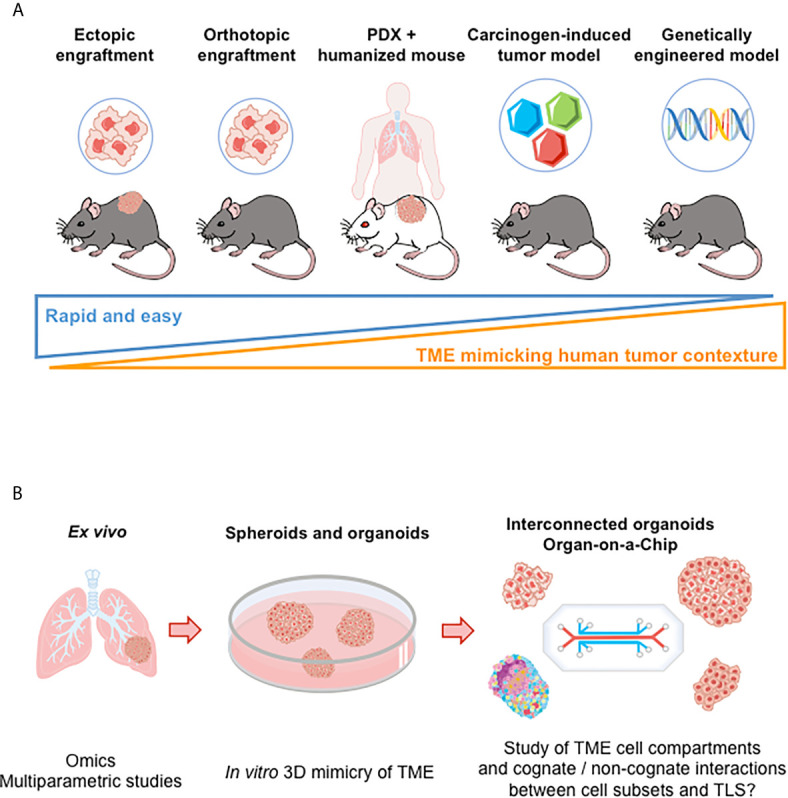
Preclinical models for the study of TLS. **(A)** Illustration of murine models to investigate of TLS neogenesis and immune function. From the left to the right: i) Ectopic models consist in implanting syngeneic tumor cells in immunocompetent mice (subcutaneous injection). However, tumor microenvironment poorly recapitulates the immune contexture of the originating tissue; ii) Intravenous injection of tumor cells allow tumor cells to disseminate in various tissues. But TLS study is hard to perform (kinetics of tumor growth and of TLS neogenesis to be mastered); iii) Implanting tumor cells directly into the tissue from which they originate (orthotopic models) allows tumor growth in a more physiological relevant microenvironment but without rapid dissemination; iv) Patient-Derived Xenotransplantation (PDX) tumor models that use immunodeficient mice enable a better maintenance of tumor heterogeneity but do not allow to investigate anti-tumor immune responses. Mice repopulated with human immune cells (“humanized mice”) can be used; v) Carcinogen-induced and genetically engineered tumor models better mimic the clinical situation. Tumors develop spontaneously and gradually in the targeted tissue, allowing for progressive immune microenvironment formation. However, an important variability in tumor development is observed (requirement for large numbers of animals to conduct experiments). **(B)** *Ex vivo* and in *vitro* models, as illustrated in lung cancer. Tumor explants enable to perform multiparametric cytometry and/or imaging, bulk or single cell RNAseq, providing data on the cellular and molecular content of TME. Spheroid and organoid cultures derived from tumor tissues allow the study of TME cellular components (or even of more complex structures such as TLS in a near future following recent progress in organoid and interconnected organoid techniques) (82). “Organ-on-a-Chips” (i.e., microfluidic tissue chips) devices make it possible to study the interaction between TME cell compartments (tumor, stromal, and immune cells). TME, tumor microenvironment.

The majority of murine models used for the induction of tumor-associated TLS are based on syngeneic systems (**Figure 3A**). It is a rather simple and economic mean for studying the cellular and molecular mechanisms underlying TLS formation. However, despite the substantial advantage of an intact immune system in mice, tumor growth rate in syngeneic transplanted models is faster than in cancer patients, and might not allow to induce a chronic inflammation usually observed in human TME where TLS neogenesis takes place.

That being said, preclinical models aimed at enhancing a *de novo* vascularization have been nevertheless useful to decipher some of the characteristics of TLS formation. Most of these models focus on lymphotoxin signaling as lymphotoxins are known to play a crucial role in secondary lymphoid organ formation and maintenance through high HEV. These molecules belong to the TNF superfamily and are composed of two main forms, LTα and LTβ that can combine in either LTα2β1 or LTα1β2 heterodimers. The latter heterodimer is the predominant form and binds to LTβ-R receptor on epithelial cells, stromal cells present in lymphoid tissues, monocytes and DC among other cell types. There has been evidence that LTα or LTβ deficiency in mice leads to severe defects in lymphoid organs development (83, 84). Several therapeutic approaches have been considered to boost HEV formation and TLS neogenesis. An anti-disialoganglioside 2 (GD2) antibody fused to LTα has promoted anti-tumor specific T cell responses in LTα^-/-^ mice bearing GD2-expressing B16 melanoma (85, 86). TLS were observed at the vicinity of the tumor, and immunohistochemistry revealed an increased tumor infiltration by immune cells and the presence of B and T cell zones adjacent to the tumor site. Targeting of LTβ-R has also proven preclinical efficacy in both syngeneic and xenogeneic models of colon carcinoma. An agonist anti-LTβ-R antibody has been shown to boost HEV development and TLS induction in a syngeneic mouse colon carcinoma model (87). This was accompanied by tumor growth inhibition and a better response to chemotherapy. Anti-tumor efficacy was also reportedly greater in orthotopic models, highlighting the fact that the growth of tumors in the tissue from which tumor cells are initially derived is an important factor for immune infiltration and TLS neogenesis.

Another ligand of LTβ-R is LIGHT, a pro-inflammatory cytokine member of the tumor necrosis factor (TNF) superfamily, also termed TNFS14, and expressed on immature DC and activated T lymphocytes. Preclinical studies have demonstrated the role of LIGHT protein in activating LTβ-R signaling pathway and TLS neogenesis in solid tumors. In a mouse model of MC38 colon carcinoma, Tang et al. demonstrated that targeting the tumor with an anti-EGFR antibody fused to mutated LIGHT protein (optimized for binding to the mouse lymphotoxin-beta receptor (LTβ-R) and herpes virus entry mediator (HVEM) receptor and for preventing the spontaneous aggregation of the recombinant LIGHT molecule) enhanced T-cell anti-tumor response and contributed to overcome resistance to PD-L1 blockade by facilitating HEV induction and T-cell tumor infiltration *via* CD68^+^ macrophages activation (88). It has been also shown that targeting tumor vessels with LIGHT fused to a vascular targeting peptide (VTP) (CRGRRST or CGKRK that have been shown to bind tumor blood vessels in murine breast, melanoma, SCC, and brain tumors), to subcutaneously implanted Lewis Lung Carcinoma (LLC) tumor cells (resistant to ICP inhibitors) induces a dose-dependent neovascularization and TLS formation, resulting in increased survival and synergistic effects with anti-tumor vaccination (89). Similar effects induced by LIGHT-VTP were observed in an immunocompetent mouse model (RIP1-Tag5 mice) where pancreatic neuroendocrine tumors arise spontaneously as well as in an orthotopic syngeneic model with the use of the CGKRK peptide as VTP fused to LIGHT after intracranial implantation of murine glioblastoma cells in C57Bl/6 mice. Intra-tumor HEV formation was enhanced, resulting in increased T cell infiltration and anti-tumor response. The effects mediated by LIGHT targeted to blood vessels were multiplied by combining CGKRK-LIGHT therapy with an anti-VEGF antibody or with ICP inhibitors (90). Interestingly, a selective binding of CGKRK-LIGHT was also observed on tumor vessels but not on normal vessels of FvB/N Rag-deficient mice implanted with human NSCG glioblastoma cells, suggesting that LIGHT-targeting strategies are translatable to human neo-angiogenic tumors.

Moreover, the role of CCL21 in the induction of TLS has been also investigated. CCL21 is a chemoattractant expressed by fibroblastic cells within lymph nodes and its role as a T lymphocyte recruiter into TLS *via* CCR7 has been extensively studied. Intra-tumor injection of the chemokine in ectopic pancreatic adenocarcinoma increased tumor immune infiltration, especially T cells, DC and NK cells, and induced a microenvironment reorganization resulting in anti-tumor immunity (91). The use of different KO mice revealed that the effect of CCL21 is mediated by these various immune cell populations and not due only to the angiostatic effect of the therapy. Also, the use of genetically engineered DC has been proven efficient in murine models. T-bet transcription factor-expressing DC induced TLS neogenesis in a model of ectopic CRC and in sarcoma (92). In these studies, TLS induction by T-bet^+^ DC was found to be dependent on IL-36γ overexpressed by these cells.

Transfer of established clonal cell lines have also been reported to induce TLS *in vivo*. In an elegant study performed by Zhu et al., lymph node-derived and isolated CD45^-^ CD3^-^ LTβ-R^+^ VCAM-1^+^ clonal stromal cells were reinjected into mice before ectopic subcutaneous MC38 colorectal cancer engrafting. Formation of TLS, accompanied by an increased infiltration of anti-tumor specific T lymphocytes was observed in pre-injected mice (93).

In a preclinical study, combination of GVAX with TGF-β inhibitors enhanced TLS formation, CD8^+^ T cells infiltration, and intra-tumor Treg depletion in pancreatic tumor-bearing mice, resulting in an improved anti-tumor effect of the vaccination (94). This pre-clinical study has been echoed in a clinical trial where the administration of GVAX was shown to induce TLS formation, promoting T cell infiltration and Th17 differentiation, while inhibiting Treg recruitment in PDAC patients (35). Anti-angiogenic strategies as well as ICP inhibitors combined with chemotherapy could also enhance immune infiltration through HEV and promote TLS neogenesis in breast and pancreatic cancer syngeneic models, although not in a glioblastoma model (95).

Other mouse models relying on the injection of syngeneic lung tumor cells in immunocompetent mice have been used to study lung tumor development and response to therapy but have rarely reported examination of TLS. The injection of mouse lung tumor cells such as syngeneic LLC in immunocompetent mice induced the appearance of a lymph node-like vasculature, supporting naïve T-cell entry into the tumors (96). It was found dependent on the presence of endogenous CD8^+^ T cells but not of B cells, NKT and CD4^+^ T cells. However, the authors also showed that cells involved in the induction of lymph node-like vasculature and cytokine production differed between tumor sites. Finally, this mouse model made it possible to show that organized lymphoid tissue develops in tumors injected intra-peritoneally, although T cells were not organized in a well-defined area.

#### Humanized Tumor Models

TLS have been successfully induced in both ectopic and orthotopic tumors from human origin representing a variety of cancer types. They were mostly detected thanks to their specific immunohistological features that include the presence of a T cell zone and an adjacent B cell zone punctuated by clusters of follicular dendritic cells, structured by a network of HEV, and a specific chemokine expression profile such as CCL21 and CXCL13. However, the existing models have limitations as they fail at reproducing the complex human TME and tumorigenesis kinetics, both of which greatly impact TLS neogenesis and function. In addition, xenografts of human tumor cells are attractive but not workable for TLS study as they require the use of immune-deficient mice which prevents to study the immune cellular crosstalk that takes place to generate TLS. The engrafting of patient-derived tumor tissue (patient-derived xenograft, PDX) in humanized immune competent mice could enable to accurately study TLS neogenesis but remains a difficult and time-consuming task (**Figure 3A**).

#### Carcinogen-Induced Tumors

Carcinogen-induced tumorigenesis allows for evolutionary cellular and molecular interactions between immune cells and tumor cells before the tumor gets palpable, an advantage rarely shared by tumor cell transplantation, if any (**Figure 3A**). In carcinogen-induced advanced tumors, Treg accumulation in TLS may alter further development and effector function of the structures. Depletion of these cells in Foxp3^DTR^ tumor-bearing mice boosted anti-tumor efficacy through HEV neoformation and immune cell infiltration (97, 98).

#### Genetically Engineered Mouse Tumor Models

Genetically engineered murine models have also been developed, marked by a slower tumor expansion and a histopathological context that better mimics human cancer progression (**Figure 3A**). However, murine tumors are also known to bear a lower mutational load than their human counterparts, and this feature could mean a lower immunogenicity, explaining the tardiness of tumor development in genetically engineered mouse models.

This has been exemplified by the elegant work of Tyler Jacks et al. that explored the role of Treg in a genetically engineered mouse model (*Kras* ^G12D^ *Trp53^-/-^*) of autochthonous lung adenocarcinoma (97). After detecting an accumulation of activated Treg strongly expressing CTLA-4 in lungs with tumor nodules, the authors showed that these cells suppress anti-tumor responses. Depletion of Treg that were engineered to specifically express diphtheria toxin receptor fused to GFP [(DTR)-GFP fusion] provoked a massive cellular infiltration into tumors whose architecture was profoundly disrupted, as shown by CLARITY 3D confocal imaging. IHC showed that most of the infiltrating cells were CD4^+^ and CD8^+^ T cells located all over lung parenchyma, with a large number of macrophages infiltrating tumor masses whereas T cells were primarily found near/within blood vessels and macrophages within airway-like pockets surrounded by tumor cells when Treg were present. Treg were found mostly in perivascular and T-cell areas of TLS. Finally, HEV present in TLS exhibited T cells in their lumen, suggesting that HEV are involved in the recruitment of circulating T cells into TLS. Not only this mouse model made it possible to characterize in details the cell composition of tumor-associated TLS but also to investigate their function and regulation. First, tumor-specific activated dye-labelled T cells entered the lung tissue only when TLS were present in tumor-bearing mice. Control mice lacking TLS did not show any T cells in lung tissue. Interestingly, a majority of tumor-specific T cells were observed within TLS by contrast to control labeled T cells. These cells interacted with DC in the T-cell area. Second, when Treg were depleted, a strong increase in the area of the lung covered by TLS was observed due to local CD4^+^ and CD8^+^ T cell proliferation shortly after Treg depletion, an upregulation of the expression of CD80 and CD86 costimulatory molecules on DC being observed as well.

In CC10-TAg mice, intrapulmonary administration of engineered CCL21-expressing DC also induced tumor T cell infiltration and specific anti-tumor responses in spontaneous broncho-alveolar carcinoma, thus decreasing tumor burden (99). These authors could show that both ectopic syngeneic or orthotopic autochthonous tumors are infiltrated by immune cell populations following CCL21 therapy, leading to a potent anti-tumor effect. As already stated in *Learning From TLS Study in Non-Tumor Inflammatory Diseases to Better Understand TLS Role in Cancer*, CCL21 could be therefore an interesting and translatable target for TLS induction and anti-tumor immunotherapy.

Other complex genetically engineered models have been developed lately to induce tumor-associated TLS in mice. Kras^LSL-G12D^TP53^flox/flox^ mice developed lung adenocarcinoma following intra-tracheal injection of non-recombinant lentiviruses expressing tumor antigenic peptide and Cre recombinase and displayed highly organized TLS along with tumor growth (100). In this experimental model, inducible neo-antigen expression provided evidence that tumor antigens play a role in TLS formation.

Ectopic lymphoid structures histologically comparable with their human counterparts have been also induced in a genetically engineered model of IKKβ(EE)^Hep^ mice. These animals show a persistent activation of the IκB kinase (IKK) in hepatocytes and abundance and transcriptional activity of NF-κB, similar to what is observed in chronic hepatitis (14). Following diethyl nitrosamine injection, these mice are more susceptible to hepatocellular carcinogenesis (HCC). A NF-κB dose-dependent TLS phenotype was demonstrated, as well as an association between TLS and hepatocarcinogenesis, and TLS were found to be a niche for HCC progenitor cells. The bad prognosis value of TLS in this type of cancer has led to the development of murine models where TLS formation is inhibited. In particular, depletion of thymocytes, NK cells and FDC in IKKβ(EE)^Hep^ mice was obtained with an anti-Thy1.2 antibody. Treated mice displayed a reduced TLS presence as well as fewer HCC. Similar results were obtained with Rag1^-/-^ mice, lacking B and T lymphocytes. This murine model underlined an unexpected role of TLS in a subtype of HCC. It also confirmed that TLS originate from a cross-talk between innate and adaptive immune system in an inflammatory context.

Although many strategies have been successful at inducing TLS in tumor models *in vivo*, only a few murine models have shown spontaneous tumor-associated TLS formation so far. Interestingly, these tumor models have revealed various mechanisms that control TLS induction: following subcutaneous tumor implantation in Lewis lung carcinoma (96), in carcinogen-induced colorectal cancer (45), in melanoma where TLS formation was shown to be orchestrated by a network of lymphoid tissue organizer cell-like fibroblasts (101), as well as in advanced gastric cancer and in lung adenocarcinoma in genetically engineered models (97, 102). In the latter, Treg ablation led to TLS formation, suggesting that targeting Treg could be an interesting therapeutic lead for a TLS-boosting strategy.

### Spheroids and Organoids, the Next Step to Study TLS

Whether mouse models mirror accurately the cellular and molecular complexity of human tumors such as NSCLC, remains an open and controversial question, even though these models have brought useful insights on TLS role and structure. One can think that the ability to recapitulate *in vitro* the complexity of TME of human tumors and its dialogue with tumor cells could allow the study of TLS formation, regulation and function in a more relevant setting. Thus, the question arises whether the current development of 3D culture models of solid tumors could make it possible to study TLS.

Tumor spheroids correspond to simple 3D structures obtained either with cells from tumor cell lines or from biopsies, digested/dissociated or not. Spheroids resemble the architecture and metabolism of the tissue of origin. They are cell aggregates that grow in 3D suspensions. Overall, their volumes are comprised between 0.5 and 1 mm^3^. For tumor study, they can be obtained from cancer cell lines or from solid tumors. Spheroids have been used to screen anti-cancer drugs and oncogenic drivers, therapeutic antibodies and immune cell infiltration and trafficking. Recently, it has been shown that lung adenocarcinoma spheroids derived from cancer cell lines can be used to perform genome-wide CRISPR screens (103). Also, tissue-derived tumor spheres (TDTS) can be derived from cells obtained from partial dissociation of tumors and contain mostly tumor cells. TDTS can be used as a reliable read-out to test chemotherapeutic drugs. Spheroids can be also obtained without tissue dissociation from tumor explants without enzymatic digestion or mechanical dissociation. They are usually termed organotypic multicellular spheroids (OMS) or more simply tumor explants. Again, these multicellular spheroids have proven useful to determine the sensitivity of tumor cells to chemotherapeutic drugs or to analyze the diffusion and distribution of the anti-EGFR antibody cetuximab. They have been also used to study the content of their immune cell compartment and the impact of an anti-PD-1 antibody (nivolumab) on immune cell localization [reviewed in (104)]. However, all these types of spheroids did not make possible to obtain more complex tumor tissue organization where TLS can develop.

One step forward towards the generation of a TME favorable to TLS development might be microfluidics. These dynamic models can mimic vascularization and have been used for antibody, soluble drugs and cell diffusion within tumor spheroids. Murine and patient-derived organotypic tumor spheroids (termed MDOTS and HDOTS, respectively) loaded into 3D microfluidic devices containing immune cells have been developed in an effort to incorporate characteristics of TME and evaluate the response to ICP blockade (105). These spheroids retained autologous immune cells. The addition of anti-PD-1 antibody in the device showed that a dose-dependent killing of MDOTS in response to the treatment. This effect required the presence of CD8^+^ T cells in the spheroids. Similarly, when HDOTS were examined, it was possible to immunophenotype a large panel of these spheroids that contain B and T cell subsets and myeloid cells. Killing and production of cytokines (IL-2, IFN-γ, TNF-α) and chemokines (CCL9, CXCL13) could be also evidenced in presence of anti-PD-1 antibody. Thus, improvement of microfluidics applied to the generation of a complex TME where immune cells are present may allow the study of TLS genesis in a near future.

Alike organotypic multicellular spheroids, organoids are obtained from tumor fragments by culture in enzyme-free culture medium that makes possible to preserve tissue integrity. By contrast to spheroids, they can then be grown *in vitro* for several weeks, although their immune components tend to decrease and disappear along the culture, preventing a detailed analysis of the microenvironment. These models mimic the cellular and architectural complexity of the TME only partially and suffer from severe limitations that hamper the generation of a more complex TME. Hypoxia and an increased production of lactic acid by tumor cells cultured in such 3D architectures play a major role in this process. However, a recent study has shown that the co-culture of tumor organoids (derived from CRC and NSCLC tumors) with autologous PBMC can generate tumor-reactive T cells (106). Using this method, the authors could show that (i) tumor-reactive CD8^+^ T cells can be induced and expanded, (ii) expanded CD8^+^ T cells are tumor-specific and not anti-self T-cells, (iii) tumor organoids can be killed by autologous tumor-reactive T cells. Thus, this study shows that co-cultures of autologous primary tumor organoids and PBMC can be used to activate and expand tumor-reactive cytotoxic T cells from peripheral blood of patients. It paves the way to generate well-characterized tumor-specific T cells that could be further engineered for immunotherapy and to decipher the mechanisms leading to sensitivity or resistance to immunotherapeutic intervention. Also, one can think that co-cultures of organoids with cells that have been identified as playing a role in TLS induction could be set up. More sophisticated organoids that recapitulate the TME cellular complexity and its machinery can be envisioned. In a recent report, Kuo and colleagues have developed human and mouse cancer models to better recapitulate TME (107). These authors developed an air-liquid interface (ALI) mouse and patient-derived in bloc tumor organoid model that maintains the architecture of TME. In this setting, the patient-derived organoids (PDO) exhibit both tumor parenchyma and myofibroblast stroma, and include tumor-specific TIL, making it possible to model immune cell responses *in vitro* (107). These PDO recapitulated the parental tumor histology although continued growth did not always maintain the TME architecture, limiting the study of TME to a few weeks. Molecular analyses revealed that TIL within PDO derived from ccRCC recapitulated the TCR repertoire of the original tumor biopsies and that the immune diversity of T, B and NK cells was conserved across PDO and fresh tumors. Importantly, when human organoids derived from NSCLC, ccRCC and melanoma patients were treated with the anti-PD-1 therapeutic antibody nivolumab, a high-grade induction of *IFNG*, *PRF1*, and/or *GZMB* transcripts was observed within organoid-sorted TIL, with a pattern of TIL activation response to nivolumab similar to that observed in clinical trials. Thus, these results show that it is possible to manipulate immune cells present in organoids, suggesting that the triggering of cells present in TME and involved in TLS neogenesis is feasible.

All these studies clearly show that progress has to be made towards rendering organoid models suitable for TME studies (82) in particular if one wants to dissect complex process such as TLS neogenesis in tumors. The study by Kuo and colleagues pinpoints several important issues (107). First, a marked difference was observed between mouse and tumor patient-derived organoids. By contrast to mouse organoids, human PDO display variable growth correlating with i) high- *versus* low-grade tumor histology, ii) quality of the tumor biopsy (acquisition delay, tumor viability, pre-/post-treatment). Mouse tumors exhibit far less heterogeneity in terms of TIL activation, proliferation and cytotoxic ability than their human counterparts. Also, the contributions of the peripheral immune system and of vascularization in the shaping and control of TME are still excluded with the present PDO. It will be certainly possible in a near future to combine such PDO with other immune components from blood or/and lymph node and/or to introduce various precursor cells such as lymphoid tissue inducer (LTi) and organizer (LTo) cells within tumor organoids. Of note, Watanabe and his colleagues (108) have recently reported that immunologically active lymphoid tissues (termed by these authors aLTs) composed of human lymphoid cells and stromal cells expressing LTβR, VCAM-1, ICAM-1 and containing several lymphoid chemokines, could be stably constructed in immunodeficient mice. The spheroids were formed as the scaffolds from the stromal cells and transplanted into renal subcapsular space of immunodeficient mice together with human PBMC absorbed in collagen sponges. After a few weeks, three-dimensional organoids containing clusters of human T and B cells and scattered DC were stably formed. When PBMC prepared from healthy donors who were vaccinated against varicella zoster virus (VZV) were absorbed in the collagen sponges, a GC-like B cell proliferation, an anti-VZV specific antibody response and a human IFN-γ production were detected in these organoids.

Such steps forward will undoubtedly help to better recapitulate the cellular complexity of TME and will make it possible to study the genesis and immune function of TLS. Thus, a lot of efforts should be now devoted to the development of more complex 3D culture systems where i) additional cell partners impacting the cross-talk between cancer cells and the immune system are included, ii) hypoxia is better controlled, and iii) the quality of tumor biopsies is improved. In the long-term, interconnected organoids allowing the study of the interplay between primary tumor and metastases and immune cell trafficking should help to set-up *in vitro* model of TLS neogenesis (**Figure 3B**).

## Artificial Intelligence, a Valuable Tool for Fundamental and Clinical Research on TLS

Deep learning has revolutionized many machine learning tasks based on machine learning in recent years such as image and video classification, speech and natural language recognition. The complexity of graphical data has imposed significant challenges on existing machine learning algorithms. In the 1950s, a symbolic Artificial Intelligence (AI) program based on mathematical symbols was developed to represent objects and their relationship. However, a number of problems were encountered, in particular about the fluidity of the management of the symbols recognized by the machine, often too complex to be integrated in the early development of AI. New AI tools were therefore created to process signals based on a rationally-designed path through a network of simulated nodes, true counterparts of the synaptic junctions existing between human neurons. Thus, today, symbolic AI has been largely replaced by IA relying on “artificial neural networks” and “deep learning”. Recently, many studies on the extension of deep learning approaches to graphical data have emerged, especially in the medical field (109).

### Deep Learning and Medicine

The first deep learning studies focused on the detection or classification of radiological or clinical lesions, and reported performance superior to conventional techniques (110). To date, applying deep learning-based medical image analysis to Computer-Assisted Diagnosis (CAD) could provide decision support to clinicians and improve the accuracy and efficiency of various processes for diagnosis and treatment (109). New tools are emerging, such as those allowing the detection of retinopathies in diabetic patients (111), enabling the analysis of images obtained by magnetic resonance imaging (MRI) of the knee (112) or in the detection of melanoma (113), as well as to the automated detection of SCC of the oral cavity (114).

Histopathology has also benefited from these technical advances. Many works have focused on the recognition of histology slides by AI mostly on the recognition of immunohistochemical stainings but also include research on the identification of cancerous invasion-related markers in many cancers (115, 116). An interesting research work currently underway is to predict the response to anti-PD-L1 antibody treatment in melanoma and lung cancer by analyzing tumor mutational burden, MSI, and PD-L1 expression using histopathological images (117).

### Deep Learning and TLS

**Figure 4:**
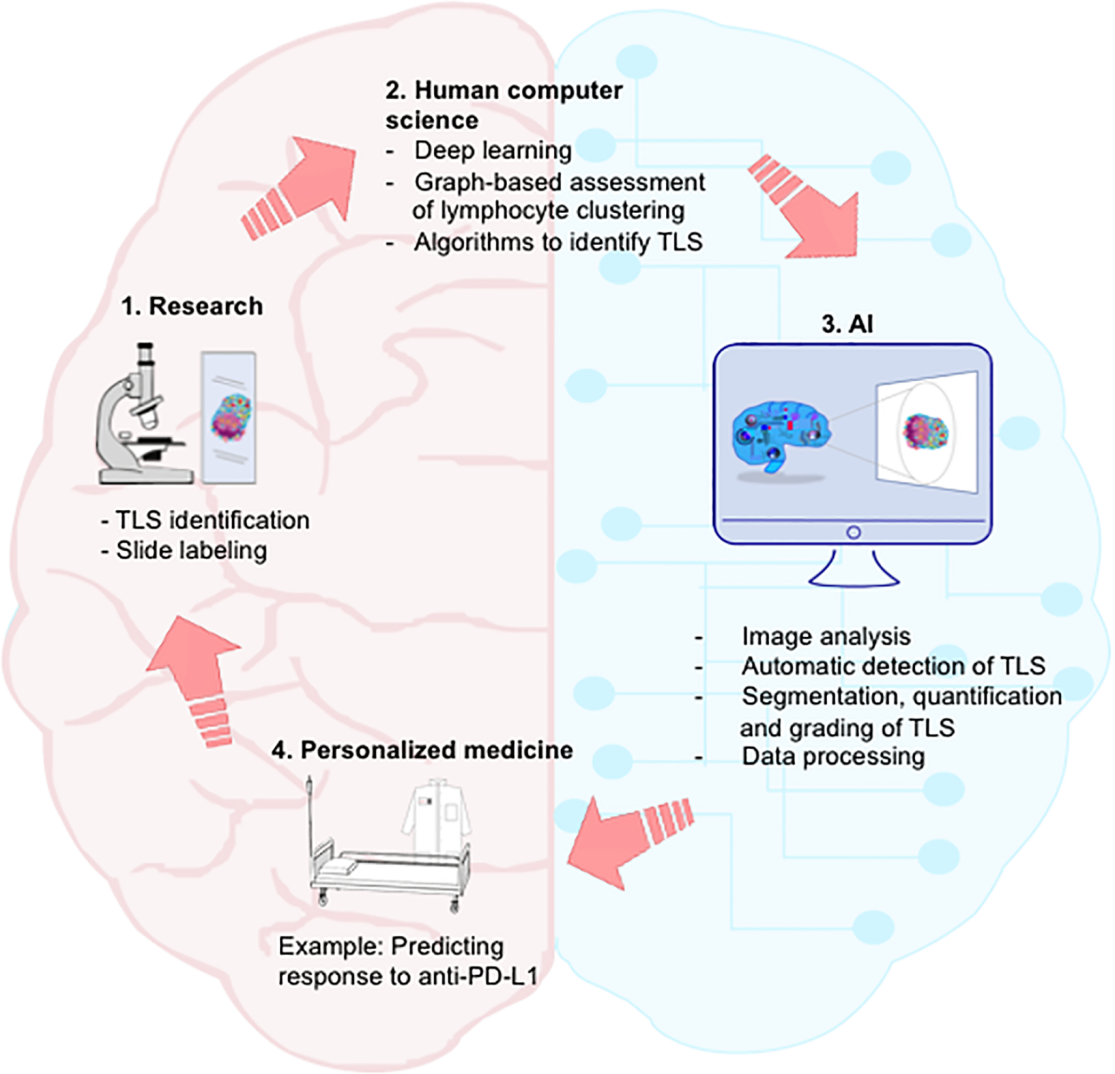
Integration of AI in the continuum of medicine. Streamlined dialogue between human and computer may accelerate path from laboratory discovery on TLS to clinical application and personalized medicine. Step 1. Researchers and clinicians identify TLS on labeled slides. Step 2. Computer scientists develop algorithms allowing machine-learning for automatic identification of TLS. Step 3. AI performs segmentation, quantification and grading of TLS, as well as big data processing. Step 4. By integrating computerized medical data into the therapeutic decision, anticipation of clinical responses (in particular following anti-PD-L1 therapy) and development of personalized medicine would become possible. AI, artificial intelligence.

Currently, a computer tool can recognize and quantify inflammatory lymphocytic infiltrates by analyzing microscope images with reproducible measurements of the spatial composition in lymphocytes. Such a computer-based reading has been developed for analyzing renal allograft biopsies, breast cancer biopsies, and lung tissue from patients with cystic fibrosis (118). It makes it possible to quantify and spatially evaluate TLS on tissue section i.e., from dense lymphoid aggregates after hematoxylin/eosin counterstaining to multiplex staining such as CD3, CD20, DC-Lamp, CD21, and PNAd co-staining (**Figure 4**). Thanks to the identification of regions of interest, the automated detection of cells of interest within the tumor stroma (in addition to the detection of stained tumor cells and/or recognition of less dense/cohesive tissue), and the classification of immune cell clusters according to their degree of organization, the computer system generates a grading of TLS for each of these diseases (118). It has also been possible to establish multi-class segmentation of tissues in breast cancer and grading of TLS in lung cancers (119).

## Conclusions and Perspective

The growing number of articles focusing on the study of TLS biology has led to a major advance in our knowledge of this singular immunological entity that arises in undedicated organs upon inflammation triggered by pathological events, including cancers.

First, apparent discrepancies in some studies highlight the importance of standardizing certain procedures and methods for the identification and quantification of TLS. Assessing several immune parameters for the determination of TLS is time-consuming and not compatible with clinical practice, highlighting the need to simplify and/or automatize their evaluation in the perspective of a TLS routine clinical protocol, in particular in human cancers. Emerging techniques of AI will certainly make it possible to standardize the recognition of TLS, leading to a reproducible reading and will therefore constitute a valuable tool for translational research and clinical practices.

It will be also necessary to find alternative identification methods that are less invasive in order to investigate TLS status, for example in cancer patients under treatment or in non-operable patients. A number of methods are currently being tested using peripheral blood or other body fluids to detect and quantify biomarkers predictive of TLS presence. In HNSCC, the analysis of saliva that is an easy-to-collect, non-invasive and easily stored fluid, could offer an alternative to facilitate the examination of tumor-associated TLS. Interestingly, this approach has already been investigated in Gougerot-Sjögren syndrome where pregnancy-associated plasma protein A (PAPPA), thrombospondin 1 and YY peptide were associated with the presence of ectopic GC that might be TLS (120).

Second, the very in-depth characterization of the cellular composition of TLS in most solid tumors has shown an heterogeneity in term of TLS organization, suggesting that the TME and tumor cells may control their neogenesis program. Deciphering the TME allowing – or not - the formation of TLS may represent a new opportunity to identify TLS inducer(s) and TLS blocker(s) that can have a direct clinical application in cancers and autoimmune diseases, respectively.

In addition, it has been shown that some cancer types are more prone to develop immature TLS (e.g. ccRCC) or mature TLS (e.g. NSCLC), whereas others exhibit mix developmental stages. Interestingly, this observation seems to correlate with response to immunotherapy. Because mature TLS are a key site where the generation of anti-tumor immunity can take place in the tumor bed, these ectopic lymphoid organizations play a dual role. On one hand, they are a powerful prognostic biomarker in almost all human cancers and on the other hand, they can predict response to ICP inhibitors (i.e., antibodies against CTLA-4, PD-1, PD-L1, or a combination). One can hypothesize that ICP blockade can reactivate protective immune responses elicited in TLS. Further investigation will be required to decipher the cellular and molecular mechanisms underlying ICP blockade efficiency as well as to extend studies to other targeted therapies. One way to understand why some patients are responders and others non-responders/progressors to ICP blockade could be through the use of spheroids and/or organoids to better recapitulate the cellular complexity of the TME and to predict the best ICP response for each cancer patient.

In conclusion, TLS are certainly an attractive target for researchers and clinicians to improve cancer patient outcome. TLS could become a new predictive biomarker of tumor development, and the capability of tumor-infiltrating immune cells to organize into TLS could be assigned as a new hallmark of cancer progression, a concept initially described by Hanahan and Weinberg (121).

## Author Contributions

CD, JR, CR, J-LT, VM, and M-CD-N wrote the initial draft. MP performed the multiplex staining. All authors contributed to the article and approved the submitted version.

## Funding

This work was supported by the “Institut National de la Santé et de la Recherche Médicale (INSERM), Sorbonne Université, Fondation ARC pour la Recherche sur le Cancer (GL: PJA2017, M-CD-N: PJA20181207895 and PGA12019120000978, J-LT: PJA20191209801), the Institut National du Cancer (INCa-DGOS_10888, M-CD-N), Cancéropôle Ile de France (2021-1-EMERG-47-INSERM 6-1), AstraZeneca (Gaithersburg, USA, n°11796A10, M-CD-N), Janssen Horizon (CT7088), and Sanofi innovation Awards Europe 2020 (201014). CR is supported by a grant from “La Ligue contre le Cancer”. MP is supported by a grant from Sanofi. CD is financially supported by Bordeaux University Hospital and Bordeaux University.

## Conflict of Interest

The authors declare that the research was conducted in the absence of any commercial or financial relationships that could be construed as a potential conflict of interest.
